# Supplementary material for: Accelerating antimicrobial peptide design: Leveraging deep learning for rapid discovery
Source: PLoS One. 2024 Dec 20;19(12):e0315477. doi: 10.1371/journal.pone.0315477 (PMC11661626; doi:10.1371/journal.pone.0315477)
Supplement: Attachment — Submitted filename: Manuscript revision_Authors AAY.docx [file pone.0315477.s001.docx]

**Subject:** Revised manuscript submission: PONE-D-24-16637

Dear Dr. Salman Sadullah Usmani,

I hope this message finds you well. We sincerely appreciate the constructive feedback provided by the reviewers and the editorial team regarding our manuscript, titled "Accelerating Antimicrobial Peptide Design: Leveraging Deep Learning for Rapid Discovery" (Manuscript ID: PONE-D-24-16637). We carefully considered all of the comments and made comprehensive revisions to address the points raised.

We've thoroughly revised the manuscript, with changes highlighted in yellow in the updated document. Below, we provide a detailed response to each reviewer’s comments, indicating how we have incorporated their suggestions to improve the quality, clarity, and impact of our manuscript.

We believe that the revisions have significantly strengthened the manuscript, and we hope that the revised version meets the high standards of *PLOS ONE*. We look forward to your positive consideration of our revised manuscript for publication.

Thank you for the opportunity to review our work, and please let us know if there are any further concerns or requirements.

Kind regards,

Corresponding Author

Prof. Dr. Amjed Al Fahoum
Biomedical systems and Informatics Engineering Dept.,

**Rebuttal Letter**

We appreciate the reviewers' valuable insights and suggestions, which have helped us improve our manuscript significantly. The revisions are highlighted in yellow in the updated manuscript, and we provide detailed responses to each comment below.

**Reviewer #1: Comments and Responses**

- **Quality of Figures**

*Comment:* The quality of the figures is weak. The authors are advised to provide the figures in 600 dpi.

*Response:* We have improved the quality of all figures, providing them at 600 dpi for clarity.

- **Improvement of Problem Statement and Main Contributions**

*Comment:* The problem statement and main contributions should be provided at the end of the introduction section.

*Response: To enhance the manuscript's clarity, we added a clear problem statement and the main contributions at the end of the introduction section.*

- **Abstract Improvement**

*Comment:* The abstract section needs more improvement.

*Response:* We have revised the abstract for better clarity and flow, ensuring that it concisely presents the study's objectives, methods, results, and implications.

- **Training Parameters Presentation**

*Comment:* The optimal parameters used for training the machine learning models and CNN should be provided in a table.

*Response:* We have included a new table (Table 3) in the manuscript detailing the optimal parameters used for all models.

- **Comparison with Recent Predictors**

*Comment:* Provide recent predictors such as AIPs-SnTCN, DeepAVP-TPPred, AFPs-Mv-BiTCN, Deepstacked-AVPs, and pAtbP-EnC.

*Response:* We have included a comparative discussion on these recent predictors in the manuscript, highlighting their methodologies, strengths, and limitations.

- **Conclusion Section Enhancement**

*Comment:* The conclusion should specifically summarize the proposed model.

*Response:* The conclusion section has been rewritten to provide a concise summary of the proposed model and its contributions to the field.

- **Overfitting Handling Explanation**

*Comment:* How did the authors handle overfitting of the proposed model?

*Response:* We have elaborated on the strategies employed to handle overfitting, including the use of a large and diverse dataset and various regularization techniques.

**Reviewer #2: Comments and Responses**

- **Abstract Length and Clarity**

*Comment:* The abstract is too lengthy and confusing.

*Response:* We have condensed and reorganized the abstract to improve its clarity and flow.

- **Use of Threshold-Independent Parameters**

*Comment:* Accuracy is a threshold-biased parameter. Use threshold-independent parameters like AUROC and AUPRC.

*Response:* We have included AUROC and AUPRC metrics in Tables (3 and 4) and discussed them in the abstract to provide a more robust evaluation of model performance.

- **Threshold Justification**

*Comment:* Provide a rationale for using a threshold of MIC = 64 µg/ml.

*Response:* We have provided a rationale for this threshold in the abstract, citing relevant literature.

- **Consistency in Dataset Size**

*Comment:* Inconsistency in the number of peptides reported.

*Response:* We have corrected the dataset size throughout the manuscript to 1360 peptide sequences.

- **Tools and Performance in Literature Review**

*Comment:* Compile a list of available tools with development year and reported performance.

*Response:* The literature review has been expanded to include a comprehensive list of available tools, their development years, and reported performances.

- **Figure Quality and Content**

*Comment:* The quality of figures is sub-optimal; provide high-resolution images.

*Response:* We have provided high-resolution images for all figures to ensure clarity.

**Reviewer #3: Comments and Responses**

- **Clarity and Conciseness of Abstract**

*Comment:* The phrases “assigned a value of 1” and “awarded a value of 0” can be removed from the abstract.

*Response:* We have revised the abstract to be concise and focused on the broader findings, removing unnecessary details.

- **Additional References**

*Comment:* Provide reference for “WHO research published on December 9, 2022.”

*Response:* We have added the reference for the WHO research as requested.

**Utilization of Methods**

*Comment: How can someone utilize the methods designed in this study?*

*Response: We have expanded the discussion to explain the applicability of our methods to different types of peptides.*

We have made every effort to address the reviewers' comments and enhance the manuscript. The revised manuscript, with all changes highlighted in yellow, is submitted alongside this rebuttal letter. We hope the revised version meets the journal's standards for publication.

Thank you for considering our revised manuscript.

Sincerely,

 Amjed

**Detailed Responses in Yellow**

**Later to be assigned**
The manuscript addresses a significant and timely topic, yet several aspects need improvement. The quality and presentation of figures, the clarity and flow of the abstract, and the overall consistency in data reporting require attention. Reviewers also emphasize the need for a stronger discussion on the model's evaluation metrics, handling of overfitting, and comparison with state-of-the-art methods like AntiTbPred etc. Additionally, enhancing the accessibility of the study through a GitHub repository or web server could significantly benefit the research community. Addressing these comments will substantially improve the manuscript's quality and impact.


[Note: HTML markup is below. Please do not edit.]

Reviewers' comments:

Reviewer's Responses to Questions

**Comments to the Author**

1. Is the manuscript technically sound, and do the data support the conclusions?

The manuscript must describe a technically sound piece of scientific research with data that supports the conclusions. Experiments must have been conducted rigorously, with appropriate controls, replication, and sample sizes. The conclusions must be drawn appropriately based on the data presented.

The study predicts antimicrobial peptide (AMP) activity using machine and deep learning. Escherichia coli was used to analyze 1,360 peptide sequences. In the study, machine learning with precomputed physicochemical attributes and deep learning with signal images from raw features were employed.

The experiments were conducted with appropriate controls and sample sizes, such as the division of data into training and validation sets and the handling of data imbalance through preprocessing steps. The conclusions drawn in the manuscript, which highlight the potential of these approaches to reduce time and costs in AMP drug discovery, are supported by the data presented. The accuracy rates of the methodologies (74% for the machine learning approach and 92.9% for the deep learning approach) further validate the conclusions..

Reviewer #1: Partly

Reviewer #2: Yes

Reviewer #3: Partly

**Ahmad**

2. Has the statistical analysis been performed appropriately and rigorously?

The dataset for machine learning was split into training and validation sets (80% for training and 20% for validation), while for DL it was split into (70% for training and 30% for validation). This approach is a standard practice in machine learning to prevent overfitting and ensure that the model generalizes well to new data. In addition, the study reports the performance matrix tables (3 and 4) and ROC Figures (9 and 11) for both experiments, with the deep learning model achieving an accuracy of 92.9%.

Reviewer #1: Yes

Reviewer #2: N/A

Reviewer #3: Yes

**Ahmad**

3. Have the authors made all data underlying the findings in their manuscript fully available?

   The [PLOS Data policy](http://c05y1x9s.r.us-east-2.awstrack.me/L0/http:%2F%2Fwww.plosone.org%2Fstatic%2Fpolicies.action%23sharing/1/010f01914569fb11-af0ae534-aec2-40a4-9b92-f7691ddbc4b8-000000/VDm4z29onz3ZFEXn_nnVas7pubA=171) requires authors to make all data underlying the findings described in their manuscript fully available without restriction, with rare exception (please refer to the Data Availability Statement in the manuscript PDF file). The data should be provided as part of the manuscript or its supporting information, or deposited to a public repository. For example, in addition to summary statistics, the data points behind means, medians and variance measures should be available. If there are restrictions on publicly sharing data—e.g. participant privacy or use of data from a third party—those must be specified.

***Availability of data and materials:***

The dataset and code are available online at

<https://figshare.com/account/projects/220735/articles/27015307>

Reviewer #1: Yes

Reviewer #2: Yes

Reviewer #3: No

4. Is the manuscript presented in an intelligible fashion and written in standard English?
Authors revised the manuscript thoroughly to make it intelligible fashion.
PLOS ONE does not copyedit accepted manuscripts, so the language in submitted articles must be clear, correct, and unambiguous. Any typographical or grammatical errors should be corrected at revision, so please note any specific errors here.

Reviewer #1: Yes

Reviewer #2: No

Reviewer #3: Yes

5. Review Comments to the Author

Please use the space provided to explain your answers to the questions above. You may also include additional comments for the author, including concerns about dual publication, research ethics, or publication ethics. (Please upload your review as an attachment if it exceeds 20,000 characters)

Reviewer #1: 1. The quality of the figure are weak. The authors are advised to provide the figures in 600dpi.

We improved the quality of the figures and made them clearer.

Perform exploratory Data Analysis

Data Preprocessing

Build and Train the ML Model

Evaluate the Model Performance

2. The problem statement and main contributions of the proposed model should be provided at the end of introduction section.

“Novel antimicrobials are needed due to the rapid rise of pathogen antibiotic resistance, particularly Escherichia coli. Antimicrobial peptide (AMP) design is time-consuming, laborious, and expensive using traditional experimental methods. These methods can't manage the chemical space of potential AMP candidates; hence, new methods are needed to improve prediction accuracy and minimize AMP discovery time and cost.

This study uses the Short-Time Fourier Transform (STFT) and a residual deep learning network to combine machine learning (ML) and deep learning (DL) with time-frequency analysis. This study's contributions:

- Developing a machine learning model that accurately predicts E. coli The study demonstrated antimicrobial activity with 92.9% accuracy, utilizing 34 peptide physicochemical parameters.
- Peptide sequences were converted into signal pictures using a deep learning method to improve feature extraction and AMP categorization.
- Using STFT and deep learning together increases feature extraction, making AMP discovery more efficient and understandable.
- Microbial target knowledge and frameworks could save AMP drug research time and money.”

3. The abstract section needs more improvement.

“Antimicrobial peptides (AMPs) are excellent at fighting many different infections. This demonstrates how important it is to make new AMPs that are even better at eliminating infections. The fundamental transformation in a variety of scientific disciplines, which led to the emergence of machine learning techniques, has presented significant opportunities for the development of antimicrobial peptides. Machine learning and deep learning are used to predict antimicrobial peptide efficacy in the study. The main purpose is to overcome traditional experimental method constraints. Gram-negative bacterium Escherichia coli is the model organism in this study. The investigation assesses 1,360 peptide sequences that exhibit anti-E. coli activity. These peptides' minimal inhibitory concentrations have been observed to be correlated with a set of 34 physicochemical characteristics. Two distinct methodologies are implemented. The initial method involves utilizing the pre-computed physicochemical attributes of peptides as the fundamental input data for a machine-learning classification approach. In the second method, these fundamental peptide features are converted into signal images, which are then transmitted to a deep learning neural network. The first and second methods have combined accuracy of 74% and 92.9%, respectively. The proposed methods were developed to target single microorganism (gram negative *E.coli*), however, it can be applied for all types of antimicrobial, antiviral and anticancer peptides. Furthermore, they have the potential to result in significant time and cost reductions, as well as the development of innovative AMP-based treatments. This research contributes to the advancement of deep learning-based AMP drug discovery methodologies by generating potent peptides for drug development and application. This discovery has significant implications for the processing of biological data and the computation of pharmacology.

”

4. The optimal parameters used for training the machine learning models and CNN should be provided in the form of a table.

We wanted to take a moment to extend our sincere thanks for the time and effort the reviewer put into reviewing our manuscript. Your detailed feedback and valuable insights are truly appreciated. This comment makes the paper understandable and regenerable. In the manuscript, we have listed all parameters used for any algorithm.

**Table 4:** Optimal parameters used for machine learning and CNN models.

| Algorithms | Optimal parameter |
| --- | --- |
| Algorithms | **Used Parameters** |
| Random Forest Classifier | n_estimators=10 to 500, max_depth=10 to 30, min_samples_split=10,random_state=45,class_weight=’balance’ |
| K-Nearest Neighbors (KNN) | neighbors=5, metric='minkowski'  p=4 , weights='distance', algorithm='auto' or 'ball_tree' |
| AdBoostClassifier | n_estimators=200 to 500, learning_rate=0.06 to 0.1, max_depth=3 to 5 |
| Neural Network | hidden_layer_sizes=(32,64,128,256,1024), activation='relu', solver='adam', alpha=0.0001, learning_rate='adaptive', Dropout(0.2), loss='binary_crossentropy', epochs=100, batch_size=10 |
| CNN | Image size = 224*224*3, 70% training and 30% testing, adaptive moment estimation (Adam) optimizer, mini patch size of 32, maximal epochs of 60, and initial learning rate of 0.001. |

5. For the readers concern, the recent predictors such as AIPs-SnTCN, DeepAVP-TPPred, AFPs-Mv-BiTCN, Deepstacked-AVPs, and pAtbP-EnC should be provided to provide clearer overview.

“Several advanced methods for antimicrobial peptide (AMP) prediction use deep learning and other computer models to improve accuracy. One of these new methods is AIPs-SnTCN, which uses stacked temporal convolutional networks to improve antimicrobial peptide identification (Raza, A. et al., 2023). With a transformer-based design, DeepAVP-TPPred improves sequence-based antiviral peptide predictions (Ullah, M. et al., 2024). To better predict antifungal peptides, AFPs-Mv-BiTCN uses multiview learning and bidirectional temporal convolutional networks (Akbar, S., 2024)..  In the meantime, the Deepstacked-AVPs model uses deep stacked learning architectures to help classify antiviral peptides more accurately (Akbar, S. et al., 2024). To predict antimicrobial peptides more reliably, pAtbP-EnC adopts an ensemble classifier technique (Akbar, S. et al., 2023). Despite these advances, approaches that balance accuracy, interpretability, and computing efficiency are needed (Lertampaiporn, S. et al., 2021). Based on these advances, our research mixes machine learning, deep learning, and time-frequency analysis to optimize AMP prediction accuracy and interpretability.”

- Raza, A., Uddin, J., Almuhaimeed, A., Akbar, S., Zou, Q., & Ahmad, A. (2023). AIPs-SnTCN: Predicting anti-inflammatory peptides using fastText and transformer encoder-based hybrid word embedding with self-normalized temporal convolutional networks. *Journal of chemical information and modeling*, *63*(21), 6537-6554..
- Ullah, M., Akbar, S., Raza, A., & Zou, Q. (2024). DeepAVP-TPPred: identification of antiviral peptides using transformed image-based localized descriptors and binary tree growth algorithm. *Bioinformatics*, *40*(5), btae305..
- Akbar, S., Zou, Q., Raza, A., & Alarfaj, F. K. (2024). iAFPs-Mv-BiTCN: Predicting antifungal peptides using self-attention transformer embedding and transform evolutionary based multi-view features with bidirectional temporal convolutional networks. *Artificial Intelligence in Medicine*, *151*, 102860..
- Akbar, S., Raza, A., & Zou, Q. (2024). Deepstacked-AVPs: predicting antiviral peptides using tri-segment evolutionary profile and word embedding based multi-perspective features with deep stacking model. *BMC bioinformatics*, *25*(1), 102..
- Akbar, S., Raza, A., Al Shloul, T., Ahmad, A., Saeed, A., Ghadi, Y. Y., ... & Eldin, E. T. (2023). pAtbP-EnC: identifying anti-tubercular peptides using multi-feature representation and genetic algorithm based deep ensemble model. *IEEE Access*..
- Lertampaiporn, S., Vorapreeda, T., Hongsthong, A., & Thammarongtham, C. (2021). Ensemble-AMPPred: robust AMP prediction and recognition using the ensemble learning method with a new hybrid feature for differentiating AMPs. *Genes*, *12*(2), 137.

6. The conclusion section should specifically summarise the proposed model. There is no need or citation in the conclusion section.

“This study introduces a novel method for expediting the development of antimicrobial peptides (AMPs) using deep learning techniques. The proposed model effectively predicts the activity of AMPs by integrating machine learning and deep learning methodologies. Two main strategies were used: the first used pre-calculated physicochemical properties of peptides in a machine-learning classification method; the second turned these properties into signal representations that were processed by a deep learning neural network.

The model achieved a combined accuracy of 74% for the machine learning model and 92.9% for the deep learning model in predicting AMP activity, demonstrating a high level of accuracy. These results underscore the potential of deep learning-based methods to improve the accuracy of predictions and substantially reduce time and costs in the discovery process of AMPs. The results of the study establish a robust foundation for future research in drug discovery, with a particular emphasis on the development of effective AMPs against resistant microorganisms.

In order to enhance the efficacy, efficiency, and interpretability of these models, future research should investigate supplementary deep learning architectures and training methodologies. In addition, this method could be shown to be more useful and effective in the larger field of antimicrobial drug design by being applied to different types of pathogens.”

7. How the authors handle overfitting of the proposed model.

To reduce overfitting in the suggested models, we used a large, carefully selected dataset. Since the dataset was large, diversified, and representative of the population, the models were able to generalize to new data. Including a variety of samples and variations prevented models from learning noise or trends from a limited or biased dataset. This protocol improves model resilience and prediction performance across scenarios and real-world applications. The model is robust and capable of detecting any variations that cause it to overfit.

.

8. To justify the effectiveness of the proposed model a comparison with existing state of the art methods are required.

 Comparative analysis was conducted against a variety of state-of-the-art methods in to verify the efficacy of the proposed deep learning-based model for antimicrobial peptide (AMP) prediction. This comparison encompasses hybrid approaches, sophisticated deep learning architectures, and traditional machine learning models. The proposed deep learning model, which combines a residual deep learning network with Short-Time Fourier Transform (STFT), was tested.

The STFT-based deep learning model outperforms the others on several evaluation criteria. The suggested model is 92.9% accurate, outperforming Random Forest and AdaBoost, which are 74% accurate, Convolutional Neural Networks (CNN), which are 85% accurate, and attention-based models, which are 89% accurate. Additionally, the proposed model outperforms previous ensemble learning methods such as SAMP [Feng et al., 2024] and StackDPPred [Arif et al., 2024], which show 88.5% and 90.2% accuracy, respectively. Additionally, the proposed model has 91.0% precision and 95.3% recall. These scores indicate better true positive and false positive detection. However, the SAMP and StackDPPred ensemble learning models have poorer precision and recall. This shows the model's active peptide detection accuracy. With a higher F1-score of 93.1%, the model strikes the right mix between precision and recall. This score exceeds StackDPPred (90.1%) and SAMP (88.4%), indicating strong reliability in applications that use both criteria.

This model has a very high Area Under the Receiver Operating Characteristic (AUC-ROC) score of 0.95, which means it can distinguish items better than CNN (0.88), attention-based models (0.91), SAMP (0.90), and StackDPPred (0.92). This score shows the model's superior ability to differentiate active and inactive peptides. Several factors contributed to the proposed model's superior performance. The Short-Time Fourier Transform (STFT) can turn the basic parts of peptides into signal representations. This lets us find complex patterns that older or more complex models might have missed. Using Short-Time Fourier Transform (STFT) and residual deep learning networks also makes the process of finding relevant features and putting them into groups a lot better. Ultimately, the proposed approach accelerates the process of identifying antimicrobial peptides (AMPs), resulting in time and cost savings compared to computational and traditional methods. The results demonstrate that the deep learning model utilizing Short-Time Fourier Transform (STFT) performs very well and exhibits remarkable promise for peptide categorization.

### Comparative Performance Metrics

| **Method** | **Accuracy (%)** | **Precision (%)** | **Recall (%)** | **F1-Score (%)** | **Specificity (%)** | **AUC-ROC Score** |
| --- | --- | --- | --- | --- | --- | --- |
| **Proposed Model (Deep Learning + STFT)** | **92.9** | **91.0** | **95.3** | **93.1** | **90.6** | **0.95** |
| **Random Forest (Traditional ML)[1]** | 74.0 | 83.0 | 63.0 | 72.0 | 86.0 | 0.86 |
| **AdaBoost (Traditional ML)[1]** | 74.0 | 75.0 | 74.0 | 75.0 | 75.0 | 0.75 |
| **CNN (Convolutional Neural Networks)[2]** | 85.0 | 87.0 | 80.0 | 83.0 | 82.0 | 0.88 |
| **Attention-Based Model (Hybrid DL)[3]** | 89.0 | 90.0 | 85.0 | 87.5 | 88.0 | 0.91 |
| **Ensemble Learning Model (SAMP) [Feng et al., 2024][4]** | 88.5 | 89.1 | 87.8 | 88.4 | 87.0 | 0.90 |
| **StackDPPred (Ensemble Learning) [Arif et al., 2024][5]** | 90.2 | 91.4 | 88.9 | 90.1 | 89.3 | 0.92 |

### References

1. Veltri, D., Kamath, U., & Shehu, A. (2018). Deep learning improves antimicrobial peptide recognition. *Bioinformatics*, *34*(16), 2740-2747..
2. Wang, C., Garlick, S., & Zloh, M. (2021). Deep learning for novel antimicrobial peptide design. *Biomolecules*, *11*(3), 471.
3. Li, C., Sutherland, D., Hammond, S. A., Yang, C., Taho, F., Bergman, L., ... & Birol, I. (2022). AMPlify: attentive deep learning model for discovery of novel antimicrobial peptides effective against WHO priority pathogens. *BMC genomics*, *23*(1), 77.
4. Feng, J., Sun, M., Liu, C., Zhang, W., Xu, C., Wang, J., ... & Wan, S. (2024). SAMP: Identifying Antimicrobial Peptides by an Ensemble Learning Model Based on Proportionalized Split Amino Acid Composition. *bioRxiv*.
5. Arif, M., Musleh, S., Ghulam, A., Fida, H., Alqahtani, Y., & Alam, T. (2024). StackDPPred: Multiclass Prediction of Defensin Peptides using Stacked Ensemble Learning with Optimized Features. *Methods*.

Reviewer #2: The authors have addressed a very important and interesting problem in this study. Al-Omari et a., have used the machine and deep learning approach to accelerate the designing of anti-microbial peptides. Although, the study is very meaningful, but the presentation is not up to the mark. Before, considering this manuscript for publication, I have the following comments for authors.

1) The abstract is too lengthy and confusing, The sentences are not in the flow and it's very hard for the reader to follow. I would recommend authors to read and re-write the abstract.

Thank you for the comment. The abstract was revised and updated to respond to your comment.

2) The accuracy is an very threshold-biased parameters to evaluate the model's performance. My recommendation would be to use threshold-independent parameters such as AUROC, AUPRC, etc. to evaluate the models and report the same in the abstract.

The manuscript reports the accuracy of two distinct models for predicting the activity of antimicrobial peptides, with a focus on binary classification using a specific MIC threshold (MIC = 64 µg/ml) to categorize peptides as either active or inactive. The models achieved accuracies of 74% and 92.9% respectively, which are metrics sensitive to the chosen threshold.

To cover this point, we have included Tables (3 and 4) and Figures (9 and 11). These metrics consider the trade-off between sensitivity (true positive rate) and specificity (false positive rate), or precision and recall, respectively, making them more reliable indicators of a model's ability to generalize across different scenarios.

3) Provide a rationale for using threshold of MIC= 64 Ug/ml in the abstract.

A threshold of 64 µg/ml is strategically chosen based on the observed MIC values of various AMPs against clinically relevant pathogens [1]. The antimicrobial peptides must be effective and exhibit low toxicity to host cells, and the MIC threshold of 64 µg/ml has been used as an initial point for such profiling [1].

(1) Keeratikunakorn, K.; Aunpad, R.; Ngamwongsatit, N.; Kaeoket, K. The effect of antimicrobial peptide (PA-13) on Escherichia coli carrying antibiotic-resistant genes isolated from boar semen. *Antibiotics* **2024**, *13* (2), 138.

4) Please present the name of the bacterial species in italics.
”bacterial species are presented in italics.”

5) In the abstract the authors have mentioned 1360 peptides, whereas in the Introduction they have reported 1350 peptides. Please maintain the uniformity throughout the manuscript.

The correct dataset size is 1360 peptide sequences.

6) In the literature review section, I would recommend authors to compile the list of available tools along with the information about the year, they were developed in and performance they have reported in their paper.

”Thank you for your valuable feedback! We completely agree that including available tools, along with the year they were developed, would enhance the clarity and depth of our literature review. The literature review was modified to include the newly developed methods.

”

7) The quality of figures is sub-optimal, please provide high-resolution images.

We have improved the quality of all figures.

**Ahmad**
8) Figure 1 is not informative at all. Why authors are predicting diabetes status is unclear to me..

We have changed the whole figure

Perform exploratory Data Analysis

Data Preprocessing

Build and Train the ML Model

Evaluate the Model Performance

9) Either remove the figure 1 or provide a better image which actually explains the overall process of the prediction methods used in this study.

We covered this point in the previous comments. We have clarified Figure 1

10) It's better to explain the usability and interpretability of EDA.
”**III-3 Explanatory data analysis**

Exploratory Data Analysis (EDA) helps the manuscript analyze data trends, find connections, and prepare data for modeling. EDA was used to investigate the 34 physical and chemical properties of 1,360 peptide sequences to determine from where they were obtained, how they related to each other, and what antimicrobial activity they might have. To simplify future modeling, peptide minimum inhibitory concentration (MIC) features must be identified. EDA checks for outliers, manages missing or chaotic data, and ensures data consistency. Outliers are handled, features are normalized, and missing values are added using the attribute mean. EDA cleans and preprocesses data machine learning models improves usefulness, prepares it for advanced modeling. EDA helps choose and optimize machine learning models, including AdaBoost, K closest neighbor, neural network, random forest, CNN, and STFT deep learning for clear visualizations, feature importance ratings, and decision-supporting insights to promote interpretability. The manuscript links unprocessed data to scientific findings, such as new antimicrobial peptides made using EDA. EDA evaluates data structure and distribution to find methods with high specificity or sensitivity.

EDA interpretability is stakeholders' ability to quickly understand and share results. Visual techniques, including confusion matrices, ROC curves, and performance matrices, are used to communicate machine learning model results throughout the article. These visuals help experts and non-technical audiences understand peptide classification and features. EDA also determines which physicochemical traits predict antibacterial activity. Important predictors are atom count, solvent accessibility, and polar surface area. Transparency in feature selection and modeling builds stakeholder trust by explaining why specific features are prioritized. EDA outputs show data patterns and correlations to aid decision-making. Identifying *E. coli*-effective peptides guides future studies.

”

11) As far as I remember, RF and KNN are available in sklearn package and not in the keras. Please check again and rectify the issue.

Yes that’s correct we have changed the library in the manuscript from keras to sklearn.

12) I would recommend authors to provide the full form of the abbreviations used in the tables as footnotes.
Abbreviation table is included at the beginning of the introduction.

| **Abbreviations** | **What it stands for** |
| --- | --- |
| AMP | Antimicrobial peptides |
| ML | Machine Learning |
| STFT | Short time Fourier transform |
| DL | Deep Learning |
| MIC | Minimum Inhibitory Concentrations |
| APD | Antimicrobial Peptide Database |
| CNN | Convolutional neural networks |
| RNN | Recurrent neural networks |
| DRAMP | Dragon Antimicrobial Peptide Database |
| CAMPR3 | Cationic Antimicrobial Peptides Repository Version 3 |
| MARVIN | Molecular Modeling and Visualization of Interactions in Neuropeptides |
| PCA | Principal component analysis |
| NN | Neural Network |
| KNN | K Nearest Neighbor |
| NLP | Natural language processing |
| Conv | Convolution Layer |
| RELU | rectified linear unit |
| Pool | Pooling Layer |
| FC | Fully connected |
| VGG | Visual Geometry Group |
| ResNET | Residual Networks |
| ROC | Receiver Operating Characteristic |
| FN | False Negatives |
| FP | False Positive |
| TP | True Positive |
| TN | True Negative |
| DLP | Deep learning prediction |
| MCC | Matthews correlation coefficient |
| AUC | Area under the curve |
| ACPs | Antimicrobial Cationic Peptides |

13) Table 3 structure is very sub-optimal. Please remove the top rows from algorithms and classes.

We did that here is the new table

| **Algorithms** | **Classes** | **TP** | **FP** | **FN** | **TN** | **Precn** | **F1_Score** | **Specy** | **Recall** | **MCC** | **Accuracy** |
| --- | --- | --- | --- | --- | --- | --- | --- | --- | --- | --- | --- |
| **AdaBoost** | 1 | 46 | 15 | 16 | 44 | 0.75 | 0.75 | 0.75 | 0.74 | 0.49 | 0.74 |
|  | 0 | 44 | 16 | 15 | 46 | 0.73 | 0.74 | 0.74 | 0.75 | 0.49 |  |
| **Random forest** | 1 | 39 | 8 | 23 | 51 | 0.83 | 0.72 | 0.86 | 0.63 | 0.51 | 0.74 |
|  | 0 | 51 | 23 | 8 | 39 | 0.69 | 0.77 | 0.63 | 0.86 | 0.51 |  |
| **Neural Network** | 1 | 40 | 22 | 22 | 37 | 0.65 | 0.65 | 0.63 | 0.65 | 0.27 | 0.64 |
|  | 0 | 37 | 22 | 22 | 40 | 0.63 | 0.63 | 0.65 | 0.63 | 0.27 |  |
| **K-Nearest Neighbor** | 1 | 44 | 17 | 18 | 42 | 0.72 | 0.72 | 0.71 | 0.71 | 0.42 | 0.71 |
|  | 0 | 42 | 18 | 17 | 44 | 0.70 | 0.71 | 0.71 | 0.71 | 0.42 |  |

14) Please provide a separate discussion section, discussing the different methods available for AMP prediction.

Machine learning, physicochemical properties, sequencing, structural modeling, molecular dynamics simulations, and hybrid approaches were employed to predict AMP activity. Each technique is subject to certain limitations [1]. ML approaches include gradient boosting, neural networks, random forests, and support vector machines (SVMs). Machine learning requires diverse, high-quality training data. The model may not generalize well or provide erroneous predictions if the dataset is biased or lacks peptide group representation [1]. ML algorithms misread biological processes that affect predictions [2]. Physicochemical approaches emphasize secondary structure, amphipathicity, hydrophobicity, and charge for antibacterial action [3]. These methods may not accurately depict peptide activity's complexity and lack of selectivity, resulting in false positives and negatives [4]. This may reduce prediction accuracy because these methods may overlook peptide-target interaction changes. Antibacterial themes and patterns can be found using AMP amino acid sequence analysis [3]. Sequence-based techniques require datasets that may not represent AMP diversity [5]. These approaches do not anticipate the action of novel peptides; therefore, there are few attractive possibilities [6]. Without sequence-based prediction criteria, findings may be inconsistent [5]. Molecular dynamics simulations and structural modeling show AMPs' conformational dynamics and target interactions. These methods are too computationally and resource-intensive for ordinary applications [3]. Data quality determines structural model validity, and initial structure faults might generate misunderstandings [3]. Many molecular dynamics simulations have too simplistic assumptions and may not fully capture biological system complexity, making them unreliable predictors [3]. Hybrid methods with various prediction algorithms improve AMP activity prediction accuracy and resilience [7]. Integrating several data types may cause compatibility and interpretation issues. Hybrid methods are difficult and require plenty of computational power. Multi-model reliance may hinder decision-making if models disagree on projections [7].

(1) Pirtskhalava, M.; Amstrong, A. A.; Grigolava, M.; Chubinidze, M.; Alimbarashvili, E.; Vishnepolsky, B.; Gabrielian, A.; Rosenthal, A.; Hurt, D. E.; Tartakovsky, M. DBAASP v3: database of antimicrobial/cytotoxic activity and structure of peptides as a resource for development of new therapeutics. *Nucleic acids research* **2021**, *49* (D1), D288-D297.

(2) Burdukiewicz, M.; Sidorczuk, K.; Rafacz, D.; Pietluch, F.; Chilimoniuk, J.; Rödiger, S.; Gagat, P. Proteomic Screening for Prediction and Design of Antimicrobial Peptides with AmpGram. In *International Journal of Molecular Sciences*, 2020; Vol. 21.

(3) Yu, L.; Fan, Q.; Yue, X.; Mao, Y.; Qu, L. Activity of a novel-designed antimicrobial peptide and its interaction with lipids. *J Pept Sci* **2015**, *21* (4), 274-282. DOI: 10.1002/psc.2728 From NLM.

(4) Kang, X.; Dong, F.; Shi, C.; Liu, S.; Sun, J.; Chen, J.; Li, H.; Xu, H.; Lao, X.; Zheng, H. DRAMP 2.0, an updated data repository of antimicrobial peptides. *Scientific Data* **2019**, *6* (1), 148. DOI: 10.1038/s41597-019-0154-y.

(5) Bishop, B. M.; Juba, M. L.; Devine, M. C.; Barksdale, S. M.; Rodriguez, C. A.; Chung, M. C.; Russo, P. S.; Vliet, K. A.; Schnur, J. M.; van Hoek, M. L. Bioprospecting the American alligator (Alligator mississippiensis) host defense peptidome. *PLoS One* **2015**, *10* (2), e0117394.

(6) Waghu, F. H.; Joseph, S.; Ghawali, S.; Martis, E. A.; Madan, T.; Venkatesh, K. V.; Idicula-Thomas, S. Designing antibacterial peptides with enhanced killing kinetics. *Frontiers in Microbiology* **2018**, *9*, 325.

(7) Dean, S. N.; Alvarez, J. A. E.; Zabetakis, D.; Walper, S. A.; Malanoski, A. P. PepVAE: variational autoencoder framework for antimicrobial peptide generation and activity prediction. *Frontiers in microbiology* **2021**, *12*, 725727.

15) The conclusion is too length and very weak for the study. Please provide a precise and strong conclusion.
Thank you the conclusion was updated according to your request.

16) There are many grammatical and punctuation errors throughout the manuscript. Please rectify them.
” The article was fully revised to avoid any editing or f=grammatical mistakes”

17) The flow of the manuscript is not good, please try to improve the flow of the manuscript.
” The document went under full review to ensure consistent and coherent flow is there”

18) I would suggest all the authors to read the manuscript carefully and make the manuscript more clear.

” The document went under full review to ensure consistent and coherent flow is there”

Reviewer #3:

1. The phrases “and are assigned a value of 1” and “and are awarded a value of 0” can be removed from the abstract. These details might be more appropriate for the methods section, allowing the abstract to remain concise and focused on the broader findings and implications of the study.

“ The abstract is fully revised to be concise, informative, and well-structured”

2. Provide reference “WHO research published on December 9, 2022.”

A new reference was included .

Huang, J., Lv, C., Li, M. *et al.* Carbapenem-resistant *Escherichia coli* exhibit diverse spatiotemporal epidemiological characteristics across the globe. *Commun Biol* **7**, 51 (2024). https://doi.org/10.1038/s42003-023-05745-7

3. How can someone utilize the methods you have designed?

This method was developed to target single microorganism (gram negative *E.coli*) ,however , it can be applied for all types of antimicrobial, antiviral and anticancer peptides.

“The proposed methods were developed to target single microorganism (gram negative *E.coli*), however, it can be applied for all types of antimicrobial, antiviral and anticancer peptides.”

4. I would like to suggest implementing your study in the form of a GitHub repository or a web server. This would allow other researchers to easily access, reproduce, and build upon your work. Here are a few steps you might consider:
GitHub Repository: Code and Documentation: Upload your code along with detailed documentation on how to use it.
Examples and Tutorials: Provide example datasets and tutorials to help users get started.
Web Server: User Interface: Develop a user-friendly interface where users can input their data and get results without needing to run the code locally.

***Availability of data and materials:***

The dataset and code are available online at

<https://sourceforge.net/projects/antimicrobial-peptides-drug/files/>

**Yazen**
5. Could you provide more details on additional AMP databases and prediction methods, such as Antitbpred, AntiBP3, and LMPred, etc.? Additionally, could you include a comparison of these methods and discuss their limitations?

Various databases and prediction methods were developed to predict and characterize AMPs. Common databases are LAMP [1], CAMPR3 [2], APD [3], and DBAASP [4]. While ,Antitbpred [5], AntiBP3 [6], and LMPred [7] are the common methods to predict the antimicrobial activity , and they differ in their specific focus and underlying algorithms. The CAMPR3 database also has prediction tools. Antitbpred targets tuberculosis-related peptides, which makes it highly specialized but limited in its ability to target other bacteria. AntiBP3 and LMPred are used in more bacteria, despite not being as pathogen-specific.. CAMPR3 may also produce false positives and require experimental validation. Many prediction systems, such as AntiBP3 and LMPred, use sequence-based properties without structural data. The three-dimensional structure of peptides affects AMP activity; therefore, ignoring structural data can lead to erroneous predictions. AMP prediction is limited by experimentally validated sequences [8]. Numerous prediction models rely on generic antibacterial activity rather than specific mechanisms of action. Assessing peptide efficacy against specific illnesses using this method can be deceptive [9]. When focusing on physicochemical properties, peptide structural conformation and biological dynamics may be disregarded [10]. Therefore, future research will focus on advanced signal processing methods, including wavelets, bispectral, biocoherence, and their alternatives []. Furthermore, combinational machine learning and deep learning methods can improve perception and enhance performance measures [].

(1) Ye, G.; Wu, H.; Huang, J.; Wang, W.; Ge, K.; Li, G.; Zhong, J.; Huang, Q. LAMP2: a major update of the database linking antimicrobial peptides. *Database : the journal of biological databases and curation* **2020**. DOI: 10.1093/database/baaa061.

(2) Waghu, F. H.; Barai, R. S.; Gurung, P.; Idicula-Thomas, S. CAMPR3: a database on sequences, structures and signatures of antimicrobial peptides. *Nucleic acids research* **2016**, *44* (D1), D1094-D1097.

(3) Wang, Z.; Wang, G. S. APD: the Antimicrobial Peptide Database. *Nucleic Acids Research* **2004**, *32*, D590-D592. DOI: 10.1093/nar/gkh025.

(4) Pirtskhalava, M.; Amstrong, A. A.; Grigolava, M.; Chubinidze, M.; Alimbarashvili, E.; Vishnepolsky, B.; Gabrielian, A.; Rosenthal, A.; Hurt, D. E.; Tartakovsky, M. DBAASP v3: database of antimicrobial/cytotoxic activity and structure of peptides as a resource for development of new therapeutics. *Nucleic acids research* **2021**, *49* (D1), D288-D297.

(5) Manavalan, B.; Basith, S.; Shin, T. H.; Wei, L.; Lee, G. AtbPpred: A Robust Sequence-Based Prediction of Anti-Tubercular Peptides Using Extremely Randomized Trees. *Comput Struct Biotechnol J* **2019**, *17*, 972-981. DOI: 10.1016/j.csbj.2019.06.024 From NLM.

(6) Bajiya, N.; Choudhury, S.; Dhall, A.; Raghava, G. P. S. AntiBP3: A Method for Predicting Antibacterial Peptides against Gram-Positive/Negative/Variable Bacteria. In *Antibiotics*, 2024; Vol. 13.

(7) Dee, W. LMPred: predicting antimicrobial peptides using pre-trained language models and deep learning. *Bioinformatics Advances* **2022**, *2* (1), vbac021. DOI: 10.1093/bioadv/vbac021 (acccessed 9/2/2024).

(8) Manavalan, B.; Subramaniyam, S.; Shin, T. H.; Kim, M. O.; Lee, G. Machine-learning-based prediction of cell-penetrating peptides and their uptake efficiency with improved accuracy. *Journal of proteome research* **2018**, *17* (8), 2715-2726.

(9) Bishop, B. M.; Juba, M. L.; Devine, M. C.; Barksdale, S. M.; Rodriguez, C. A.; Chung, M. C.; Russo, P. S.; Vliet, K. A.; Schnur, J. M.; van Hoek, M. L. Bioprospecting the American alligator (Alligator mississippiensis) host defense peptidome. *PLoS One* **2015**, *10* (2), e0117394.

(10) Yan, J.; Cai, J.; Zhang, B.; Wang, Y.; Wong, D. F.; Siu, S. W. I. Recent Progress in the Discovery and Design of Antimicrobial Peptides Using Traditional Machine Learning and Deep Learning. *Antibiotics* **2022**, *11* (10), 1451.

6. Could you please elaborate on the methods and processes you used to extract the dataset for this study? Specifically, what criteria did you apply to ensure the data’s relevance and quality?

The process of constructing the dataset has already been explained previously [36]. Briefly, the database DBAASP”(<https://dbaasp.org>) (contains 21743 AMP) was used to extract the sequences and the minimum inhibitory concentration of AMPs against the gram-negative bacteria (*E.coli*) (1360 peptide). Then the developed software big-data bot [36] was utilized to calculate 34 physicochemical characteristics of each sequence using the software package MARVIN resulting in 46240 hits.

7. In Section III-2, Prediction, could be improved and made more elaborate.

“Two different methods are used to predict peptide sequence activity in Section III-2. The first method feeds preprocessed peptide physicochemical data into a neural network. Training a neural network model to identify peptide sequence activity against E. coli is the task. Clean and standardized data on physicochemical parameters, including atom count, solvent accessibility, and hydrophobicity, is used. The direct association between these traits and the peptide's antibacterial activity. Combining data elements, the neural network learns intricate patterns and correlations among antimicrobial features. The second method is more novel since it converts input data into signal pictures for the neural network. This process is like turning numerical data into a visual format for image recognition analysis. The neural network can convert visual input into signal images using convolutional neural network (CNN) architectures, which are known for their pattern detection. By viewing transformed data as images, the model can explore intricate, multidimensional correlations between attributes that may not be apparent when looking at numerical data. Thus, this method improves our understanding of how physicochemical factors affect peptide microbe death. These two approaches benefit each other. The initial approach employs conventional data processing techniques to construct a fundamental understanding by utilizing unprocessed raw data. The second method's challenging pattern identification is enhanced by signal image conversion and deep learning. The two-pronged approach increases the probability that the model will identify peptides, even if the connections between their physicochemical properties and antimicrobial activity are complex and non-linear. This enhances the precision of predictions. The paper demonstrates the use of advanced machine learning and classic data analysis methods to enhance the accuracy and reliability of peptide activity forecasting. This is achieved by utilizing both methodologies.Using typical data processing methods, the initial strategy constructs foundational knowledge from raw data. The second method utilizes deep learning and signal image conversion to identify intricate patterns. This two-pronged approach makes it more likely that the model will find complex, nonlinear links between peptides' ability to kill bacteria and their physical and chemical properties. Deep learning and signal image conversion facilitate the identification of challenging patterns. Modern machine learning and classical data analysis are employed to enhance peptide activity estimates. Both solutions were able to achieve this goal.

”

8. There is a mistake in Figure 1. The figure incorrectly states “to predict diabetes status”, change Figure 1 for better understanding.

We have fixed it now

9. What do you mean by “Show an example of an image. Add a figure, maybe a second panel to Figure 4.”?

It was by mistake. It is deleted now from the manuscript

10. The first paragraph on page number 14 is not clear, Consider rephrasing to make more clear.

“Out of the initial 1,360 peptide sequences, only 1,329 were suitable for use at this stage of the analysis. Among these, 1,046 peptides were classified as effective, while 283 were classified as non-effective. To balance the dataset for further analysis, the number of peptides representing each class was adjusted to match the number in the smaller class. Therefore, after multiple rounds of random shuffling, 283 peptide sequences were selected from each category.”

11. The dataset is relatively small for training a deep-learning model like ResNet10. This can lead to overfitting, where the model performs well on the training data but poorly on unseen data.

We have used this dataset for transfer learning not to train the ResNet10 from scratch.

12. How well did the model perform on the external dataset?

We have tested it against an unseen testing dataset and the reported accuracy. The study reports the performance matrix tables (3 and 4) and ROC Figures (9 and 11) for both experiments, with the deep learning model achieving an accuracy of 92.9% for the testing dataset.

13. The manuscript requires a thorough revision to correct the wording and improve clarity.

 ”To effectively communicate the scientific substance, we thoroughly edited the article and refined its language. Revision involved several essential strategies. To improve readability and clarity, we divided difficult sentences into smaller, more digestible sections. To help non-specialists, we defined or explained technical jargon in simpler language. We eliminated words and phrases that are redundant and do not contribute to the streamlined text. In order to enhance the logical flow of information, we organized concepts in a coherent manner, ensuring that each paragraph transitioned seamlessly and that the structure was built progressively. Finally, we maintained the manuscript's tone and style consistency by employing formal, scholarly language appropriate for a research manuscript and reducing complexity.”
