# Supplementary material for: Accelerating antimicrobial peptide design: Leveraging deep learning for rapid discovery
Source: PLoS One. 2024 Dec 20;19(12):e0315477. doi: 10.1371/journal.pone.0315477 (PMC11661626; doi:10.1371/journal.pone.0315477)
Supplement: Attachment — Submitted filename: Responses point by point.docx [file pone.0315477.s002.docx]

Journal Requirements:


The manuscript presents promising research and is well-supported by the reviewers. However, while finalizing, I noticed a few issues that need to be addressed before it goes for the publication.
1. Reference accuracy:
o While describing the AntiTbPred method, the authors incorrectly referenced it. Instead of AntiTbPred (PMID: 30210341), they cited AtbPpred, which is a different tool developed for a similar purpose. Authors must ensure they cite the correct reference and accurately use the tool's name, "AntiTbPred," following its original capitalization and spelling conventions. Please be sure for other tools too.

***Thank you for your valuable feedback. We apologize for the confusion in referencing the tools. We found that we incorrectly cited AtbPpred instead of AntiTbPred (PMID: 30210341). We have now corrected this error and ensured that the tool names are consistently referenced with the correct capitalization and spelling conventions throughout the manuscript. Additionally, we have carefully re-checked all other tool citations for accuracy and consistency. The necessary revisions have been made accordingly.***
2. Reference formatting:
o In the literature review section, references 51 to 56 have been cited inconsistently, using both numerical and "Name et al." formats. Please revise the manuscript to ensure consistency, adhering to the PLOS ONE guidelines for referencing.

***Thank you for pointing out the inconsistency in our referencing format. We apologize for the oversight. We have revised the manuscript to ensure that references 51 to 56 are cited consistently, adhering to the PLOS ONE guidelines for referencing. All citations are now in the appropriate format as per the journal’s requirements.***
3. Relevance of references:
o The reference list appears to include several irrelevant citations. Please revise and retain only those references directly supporting the study's context and findings.

***Thank you for your helpful comment. We have carefully reviewed the reference list and removed the citations that are not directly relevant to the study’s context and findings. Only the references that directly support the research and its conclusions have been retained***
4. Consistency in naming:
o Ensure consistent formatting of E. coli throughout the manuscript, as discrepancies have been noted.

***Thank you for your feedback. We have carefully reviewed the manuscript and ensured consistent formatting of E. coli throughout the text.***
5. Claims in the abstract:
o The final sentence of the abstract claims that the methods can be applied to "all types of antimicrobial, antiviral, and anticancer peptides." This assertion is overly broad and not fully substantiated by the presented work. Please revise to better reflect the scope of the findings.

***Thank you for your insightful comment. We agree that the statement in the abstract was overly broad. We have revised the final sentence to better align with the data presented and more accurately describe the potential of the study.***

6. Clarity in results:
o The phrase "combined accuracy of 74% and 92.9%" in the abstract is unclear. Please rephrase this sentence to explicitly distinguish between the accuracies of the two methods.

***Thank you for your helpful suggestion. We clarified the sentence***
